# The Meioflume: A New System for Observing the Interstitial Behavior of Meiofauna

**DOI:** 10.1093/iob/obae016

**Published:** 2024-05-25

**Authors:** W M Ballentine, K M Dorgan

**Affiliations:** School of Marine and Environmental Sciences, University of South Alabama, Mobile, AL 36688, USA; Dauphin Island Sea Lab, Dauphin Island, AL 36528, USA; School of Marine and Environmental Sciences, University of South Alabama, Mobile, AL 36688, USA; Dauphin Island Sea Lab, Dauphin Island, AL 36528, USA

## Abstract

Meiofauna (benthic invertebrates < 1 mm in size) facilitate sediment biogeochemical cycling, alter sediment microbial community structure, and serve as an important trophic link between benthic micro- and macrofauna, yet the behaviors that mechanistically link individuals to their ecological effects are largely unknown. Meiofauna are small and sediments are opaque, making observing the *in situ* activities of these animals challenging. We developed the Meioflume, a small, acrylic flow tunnel filled with grains of cryolite, a transparent sand analog, to simulate the *in situ* conditions experienced by meiofauna in an observable lab environment. The Meioflume has a working area (28.57 mm × 10.16 mm × 1 mm) that is small enough to quickly locate fauna and clearly observe behavior but large enough that animals are not tightly confined. When connected to a syringe press, the Meioflume can produce low velocity flows consistently and evenly across the width of its working area while retaining the contents. To demonstrate its functionality in observing the behavior of meiofauna, we placed individual meiofaunal animals (a protodrilid annelid, a harpacticoid copepod, and a platyhelminth flatworm) in Meioflumes and filmed their behavioral response to a sudden initiation of porewater flow. All animals were clearly visible within the flume and could be observed responding to the onset of flow. The design and construction of the Meioflume make it an accessible, affordable tool for researchers. This experimental system could be modified to address many questions in meiofaunal ecology, such as studying behavior in response to chemical cues, allowing us to observe meiofaunal behaviors to better understand their ecological effects.

## Background

Meiofauna, benthic invertebrates between 64 µm and 1 mm, are a diverse, abundant, and ecologically important component of the marine benthos that affect many sedimentary processes at the population and community scale (Giere 2009). The presence of meiofauna affects solute transport in surficial sediments (Aller and Aller 1992; Rysgaard et al. 2000; Berg et al. 2001), enhancing biogeochemical cycling, e.g., doubling the rates of denitrification (Bonaglia et al. 2014), altering degradation pathways of hydrocarbons (Näslund et al. 2010), and stimulating organic matter mineralization by up to 50% (Nascimento et al. 2012). Meiofauna link micro- and macro-benthic fauna in marine food webs by consuming a variety of carbon sources (i.e., microalgae, protozoans, other meiofauna, bacteria, and organic detritus) then providing a high-quality food source for macrofauna (Montagna 1984; reviewed by Schratzberger and Ingels 2018). Understanding of the mechanisms underlying these processes is limited by a lack of direct observations of meiofaunal behaviors *in situ*, e.g., predator–prey interactions, feeding rates, and foraging strategies within the sedimentary environment.

The lack of observational data on the interstitial behaviors and activities of meiofauna is largely due to their size and habitat. The small size of meiofauna necessitates the use of a microscope, making *in situ* observations impractical. Natural sediments can be examined in the lab, but because of sediment opacity, observations are restricted to surface layers (or bottom layers if using an inverted microscope). Past studies of surface and bottom layers provided some of the earliest observations of meiofauna feeding in sediment, but required many hours of labor and cited the opacity of sediment as a “major stumbling block to good observations of meiofauna behavior” (Watzin 1985). As such, the first step in most meiofaunal studies is to separate the fauna from the sediment. This allows for live or dead fauna to be studied in detail beneath a microscope without being obscured by grains of sand (Giere 2009), enabling the study and documentation of meiofaunal morphology, anatomy, taxonomy, and systematics (recently compiled in Schmidt-Rhaesa 2020). However, separation of fauna from sediment impedes observations of natural behavior. Studies examining the ecology, distribution patterns, and ecosystem functions of meiofauna have largely been done without observations of interstitial activities (Giere 2009).

Transparent analogs to sands provide a potential solution to the problem of observing meiofaunal behavior. Several previously used transparent analogs have achieved differing levels of environmental realism. Coineau and Coineau (1979) manufactured cast resin pillars of varying diameters laid out in a matrix-like pattern similar to a peg board. The spaces between the pillars formed interconnected channels and, when covered with a glass slide, created a two-dimensional interstitial environment (Coineau and Coineau 1979). Although live meiofauna could be visualized in this system and the size of the interstitial channels could be customized, it lacked three-dimensional complexity, and the gridded peg-board layout poorly replicated the complexity of natural sands. Giere and Welberts (1985) improved upon this method by replacing the peg board patterning on the resin cast with interstitial channels copied from naturally mixed sand (Giere and Welberts 1985). This method more closely replicated the interstitial geometry of natural sediments but lacked the three dimensionality and environmental complexity of the natural environment. To investigate how some meiofaunal annelids utilize their palps in interstitial spaces, Ballentine and Dorgan (2023) used the mineral cryolite to create a transparent interstitial environment without porewater flow. Cryolite (Na_2_AlF_6_) is a naturally occurring, translucent, halide mineral with an index of refraction very similar to that of seawater (1.338 and 1.339, respectively) (Josephson and Flessa 1972). These unique optical properties render cryolite nearly completely transparent when submerged in seawater. Cryolite has been previously used to observe the burrowing behaviors of macrofauna (Josephson and Flessa 1972; Francoeur and Dorgan 2014; Dorgan 2018), in live cell imaging of soil microorganisms (Sharma et al. 2020), and to investigate the oxygen consumption, spatial patterns, and swimming behaviors of bacteria (Oates et al. 2005; Zhu et al. 2014).

Cryolite creates a transparent interstitial environment that makes observations of meiofaunal activities possible. Increasing the complexity of the cryolite system to better simulate natural sediments would allow for more intricate investigations of meiofauna form and function. When considering marine sands, a natural addition to this system is porewater flow. Porewater flow through interstitial spaces of marine sands is driven by pressure gradients created by overlying waves, currents, and tides (Huettel and Webster 2001; Janssen et al. 2005). These interstitial flows establish and maintain chemical gradients, replenish oxygen, transport organic matter, and distribute chemical cues, which may be important for mate location, feeding, and predator avoidance (Riedl and Machan 1972; Riedl et al. 1972; Billerbeck et al. 2006; Giere 2009).

Here, we describe and validate the Meioflume, a 7.62 cm × 2.54 cm × 1.3 cm flow chamber that we use with cryolite to observe the behaviors of interstitial fauna under environmentally relevant conditions, specifically the addition of porewater flow (0.005–0.1 cm/s). Additionally, we provide a protocol for use of the Meioflume, an open source, detailed, CAD file of its design, and a brief description of its construction. The methods described here rely heavily on the use of the mineral Cryolite that is no longer commercially mined and difficult to procure. Cryolite can be replaced in this system with other transparent analogs to sand that we discuss further below.

## Meioflume development and design

**Fig. 1:**
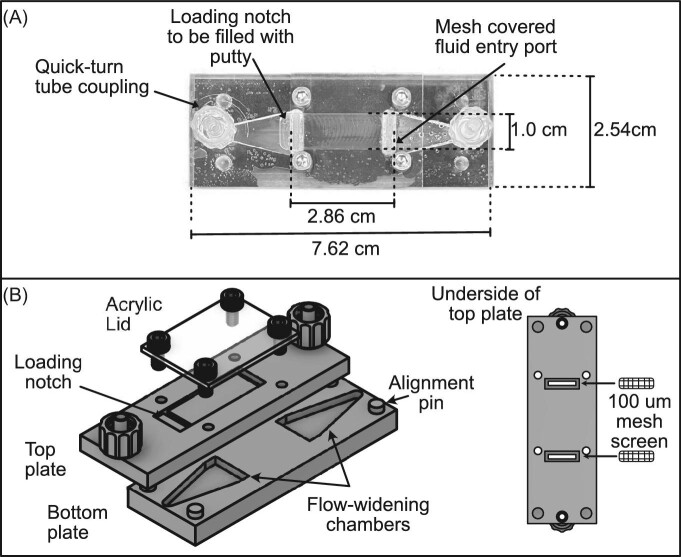
The Meioflume. (A) A manufactured and assembled Meioflume made of extruded acrylic (**B**) Deconstructed design models of the two component pieces of the Meioflume with the bolted acrylic lid shown.

**Fig. 2:**
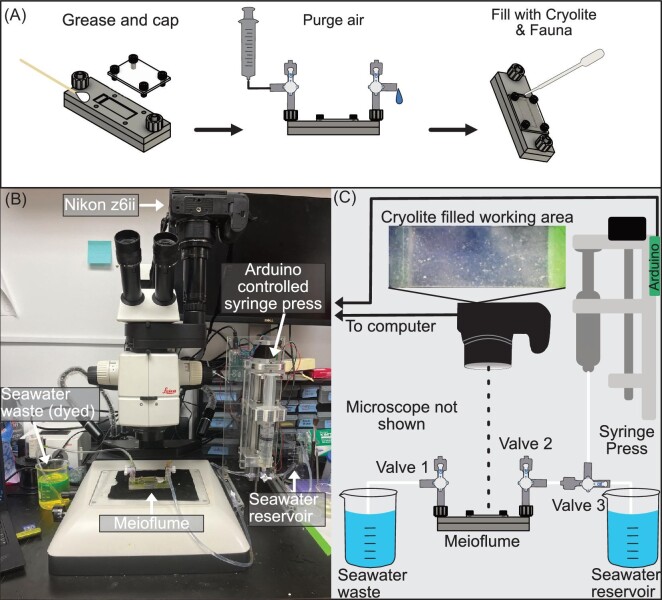
The Meioflume experimental set-up. (A) The steps required to prepare the Meioflume for use. The Meioflume is first greased, capped and sealed, then purged of air using a syringe, and finally filled with Cryolite and fauna. (B) The final version of the Meioflume on the stage of a microscope. The right side of the Meioflume is connected to the custom-built syringe press via three-way stop cock valves and tubing, while the left side is similarly connected to a waste seawater reservoir. The syringe press draws water from a seawater reservoir and dispenses it through the Meioflume. An additional three-way valve controls whether the syringe press is connected to the Meioflume or the seawater reservoir. (C) A simplified diagram of the experimental setup. To simplify the diagram, the microscope is not pictured in the diagram.

The Meioflume was developed over a series of iterative designs and tests, all of which informed the final design presented. A narrative description of our design and development process is available in the supplement. Our design goals were: A working area small enough to find and observe fauna but large enough so that fauna could locomote in three dimensions, even flow across the working area, and ease of use for repeated behavioral observations. These criteria determined the final design and dimensions (Fig. 1) of the Meioflume. The working area dimensions (2.85 cm × 1.02 cm × 0.1 cm) can be visually scanned in less than two FOV's at 10× magnification while allowing meiofauna (0.0064–0.1 cm in size) ample room for movement. Even flow is generated across the working area by the internal flow-widening chambers within the Meioflume (Fig. 1B). Fluid enters the device through one of two quick-turn tube couplings located at both ends of the Meioflume and flows into the flow-widening chamber housed within the Meioflume beneath the working area (Fig. 1B). Within the flow-widening chamber, the jet of incoming fluid gradually expands to the width of the overlying working area, then flows up through a 100 µm mesh screen (Fig. 1B) into and across the working area. The mesh screens prevent washout of fauna and cryolite from the working area. Ease of use is achieved through the Meioflume's greased and bolted acrylic lid and its loading notch/putty plug (Fig. 1). The greased and bolted acrylic lid is installed before introducing cryolite or fauna to the Meioflume, producing a robust grease seal that seals the entire working area. The loading notch extends past the acrylic lid (Fig. 2), and enables addition of cryolite and fauna to the working area after the lid has been installed. Once the working area is filled with cryolite and fauna, the loading notch is sealed using putty. More details on loading and using the Meioflume below.

### Fabrication

A full CAD file and parts list for the final version of the Meioflume can be found at (https://github.com/Will-Ballentine/Meioflume_Designs.git) (Table S3) and the fabrication process is briefly summarized. The Meioflume was milled from clear, extruded acrylic using a Tormach 1100M CNC router. It was milled as three separate pieces (Fig. 1B): (1) the lower plate that houses the flow widening chambers and four alignment pins on its surface (Fig. 1B) and (2) the upper plate, which on its surface contains the working area, the threaded ports for the quick-turn tube couplings, and the four bolt holes for the acrylic lid (Fig. 1B) (The underside of the upper plate contains two channels connecting the flow widening chamber to the fluid entry ports, and four alignment holes (Fig. 1B)); and (3) the acrylic lid with its four bolt holes.

After milling, the underside of the upper plate was fitted with 100 µm mesh screens (Fig. 1B), which are bonded in place using standard cyanoacrylate super glue. The upper plate was then permanently bonded to the lower plate using WELD-ON acrylic #4 acrylic bonding agent and allowed to set for 72 h. The alignment pins present on the surface of the lower plate (Fig. 1B), and the corresponding alignment holes present on the underside of the upper plate (Fig. 1B) ensure the accurate alignment of the flow-widening chambers and the working area. Once bonded, the quick-turn tube couplings are installed in their respective ports, and the acrylic lid can be greased and bolted into place.

### Preparation and use

To prepare the Meioflume for use, the acrylic lid must be sealed and secured, the flow-widening chambers and working area must be purged of air, and the working area must be filled with cryolite (or transparent alternative) and meiofauna. To seal and secure the acrylic lid, we first apply a thin layer of Dow-Corning High Vacuum Grease using a cotton-tipped applicator to the surface of a clean, dry, Meioflume, fully surrounding the working area (Fig. 2A). We place the acrylic lid over the working area, aligning the bolt holes, then secure it in place using four stainless steel bolts. To purge the flow-widening chambers and working area of air, we attach three-way stop cock valves to the quick-turn tube couplings and attach a 60 mL syringe of seawater to one of the open ports. Using the syringe, air is purged from the Meioflume by rapidly filling the chambers with seawater. This can be done while the device is submerged in a reservoir of seawater to increase efficiency. Once purged, the flow-widening chambers are sealed using the stop cock valves, and the syringe is removed.

To fill the working area with cyolite and fauna, the desired amount of cryolite is placed in a small dish with seawater and thoroughly wetted. The exact amount of cryolite used depends on grain size and the level of compaction desired, but we have found that ∼0.4–0.5 g of 500 µm cryolite is sufficient to fill the working area. The volume of water added is arbitrary but should be enough that the cryolite can be easily pipetted. Once the dish of cryolite and seawater is prepared, the Meioflume is tilted ∼45° so that the loading notch is above the working area (Fig. 2A). Using a transfer pipette, some of the cryolite seawater slurry should be pipetted into the loading notch in small batches, allowing gravity to fill the working area from the loading notch. Once the working area is filled to the desired level, the loading notch is sealed with putty. We have used both modeling clay and cast-silicone plugs to seal the loading notch and have found that both work well. To include meiofauna trials, place the fauna in the dish of seawater and cryolite before filling the working area and allow the animals to acclimate for several minutes, then fill the working area as described above. When physically disturbed, meiofauna will rapidly adhere to the nearest sediment grain (Giere 2009). We exploit this behavior to simultaneously fill the working area with cryolite and meiofauna.

Using the Meioflume requires a microscope and a method for generating fluid flow. The Meioflume will fit on the stage of most compound and dissection microscopes. Small-scale fluid flow can be generated with many commercially available devices; however, in our development and testing, we used a custom built syringe press controlled using an Arduino Uno microcontroller (Cho et al., in review for detailed DIY instructions on construction). This syringe press allowed for extreme flexibility in flow regimes and accurately generated flow rates ranging from 0.0001 to 1 mL s^−1^. Our experimental system consisted of a Leica M-125 dissection microscope, an attached Nikon z6ii digital camera, the custom-built syringe press, an artificial seawater reservoir that provided oxygenated water to the syringe press, a waste seawater reservoir, and the Meioflume (Fig. 2).

To fill the syringe press, valve 3 (Fig. 2C) must be set such that the port leading to the Meioflume is sealed. The syringe press can then draw from the reservoir. Once the syringe press is filled, valve 3 (Fig. 2C) is then set such that the port leading to the reservoir is sealed and the pump is connected to the Meioflume. Stop cock valves 1 and 2 are then both opened (Fig. 2C), and the syringe press is activated. Fluid flows from the syringe, through the Meioflume, and is caught in the waste reservoir (Fig. 2 B and C), while the working area is recorded using the microscope/camera.

## Calibrating and validating the Meioflume

### Calibration

A primary goal in developing the Meioflume was to simulate the small-scale flow environment experienced by meiofauna. Therefore, porewater velocity within the Meioflume should be comparable to *in situ* velocities. We know of no studies that have measured horizontal fluid velocity in marine sediments, but vertical velocities have been studied in the context of porewater nutrient exchange and range from 40 to 50 cm h^−1^ (0.111–0.139 mm s^−1^) (Precht and Huettel 2004; Reimers et al. 2004). Horizontal porewater velocities are likely to differ from vertical velocities, but without other measurements, we used vertical velocity as a mid-range target for Meioflume flow velocities. Measuring and repeating a target velocity within the Meioflume requires calibrating porewater velocity to the programmable flow rate of the syringe press. Fluid is dispensed into the system as a flow rate (*Q*, mL s^−1^), which can be used to calculate the average fluid velocity ($\bar{u}$, cm s^−1^) by dividing the flow rate by the cross-sectional area (*A*, cm^2^) of the flow field such that


(1) \begin{eqnarray*}
\bar{u}{\mathrm{\ }} = {\mathrm{\ }}\frac{Q}{A}.
\end{eqnarray*}

**Fig. 3:**
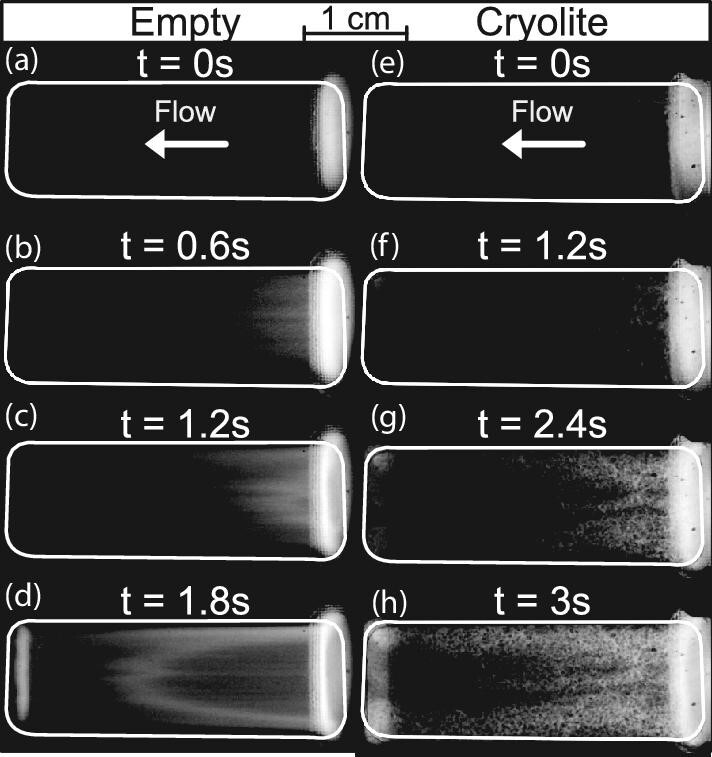
Flow through empty (A–D) and cryolite filled (E–H) Meioflumes. Flow crosses from right to left as indicated by arrows. In both trials, fluorescein dye fills the priming chamber (A and E), enters the working area of the Meioflume (B and F), moves across the working area (C and G), and finally fills the entire working area (D and H). Working area outlined in each panel.

While this is easily applied to an empty Meioflume with a known cross-sectional area (*A*), packing the Meioflume with cryolite transforms the working area from a single open channel into a matrix of small tortuous pores (Fig. 3E–H), reducing the cross-sectional area available for flow and increasing average velocity. Fluid velocity through a tunnel of known cross-sectional area filled with cryolite can be better represented by


(2) \begin{eqnarray*}
\bar{u}{\mathrm{\ }} = {\mathrm{\ }}\frac{Q}{{A*\beta }},
\end{eqnarray*}

where $\beta $ is the porosity of the granular material.

To calibrate the packed Meioflume for velocities both above and below measured *in situ* velocities, we selected our initial flow rates using the cross-sectional area of the Meioflume (0.1 cm^2^) and Equation (1). We generated porewater flow through an empty Meioflume and one packed with cryolite at flow rates of 0.0005, 0.001, and 0.01 mL s^−1^, which correspond to calculated average velocities in an empty Meioflume of 0.05, 0.1, and 1.0 mm s^−1^. To measure the porewater velocities generated by these flow rates, 0.5 mL of fluorescein dye mixed with DI water (200 mg L^−1^) was injected into the flow-widening chamber of the Meioflume prior to the initiation of flow (Koehl 2024). DI water was then dispensed at one of the above flow rates and the working area was illuminated using blue light (wavelength 395–400 nm) to excite florescence in the dye. The progression of the dye across the working area was recorded using a Nikon Z6II digital camera (1080p, 29.97 fps) attached to a Leica M125 microscope using a camera adapter from Martin Microscopes (experimental set up shown in Fig. 2B and C, blue lights not shown). Flow velocity was measured from dye progression across the working area, which was tracked in MATLAB by isolating the green color channel. Because the tip of the dye plume represents the fastest fluid velocity in the tunnel, we chose to estimate velocity by tracking the dye plume where the plume width reached 25% of the width of the working area. The time required for the dye to cross the working area was divided by the length of the working area to calculate the fluid velocity. Measurements were replicated ∼5 times for each flow rate in the empty Meioflume trials, and ∼5 times in the cryolite trials. Each cryolite trial was independently packed before use.

To determine the relationship between flow rate and fluid velocity within the Meioflume, we used a weighted linear regression on both the empty and cryolite flume trials. A weighted linear regression was selected because fluid velocity variance increased proportionally with flow rate, causing heteroscedasticity within our data set. This is not surprising given our flow rates spanned several orders of magnitude (0.0005–0.01 mL s^−1^).

**Fig. 4:**
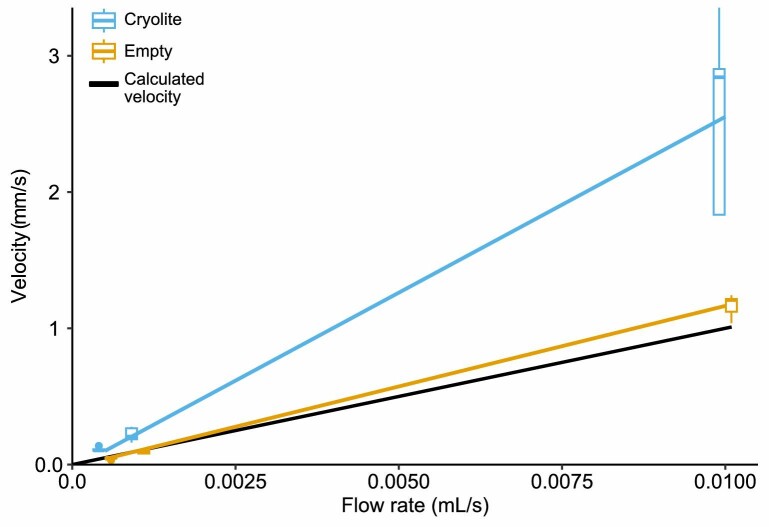
Measured velocities vs. flow rate for cryolite (*n* = 5) and empty (*n* = 5) flume trials. Lines represent weighted linear regressions fitted to each flume group. In cryolite, for every 0.01 mL/s increase in flow rate, we observed a 2.58 mm/s (±0.35; 95% CI) increase in fluid velocity (*P* = 3.15e−10, *r*^2^ = 0.94). In empty Meioflumes, for every 0.01 mL/s increase in flow rate, we observed a 1.16 mm/s (±0.046; 95% CI) increase in fluid velocity (*P* < 1e−16, *r*^2^ = 0.99).

Fluid velocities in both the empty and cryolite-filled Meioflumes were greater than the theoretical average velocities calculated using Equation (1) (Fig. 4). In the empty Meioflume trials, for every 0.01 mL s^−1^ increase in the flow rate, we observed a 1.16 mm/s (±0.046; 95% CI) increase in the fluid velocity (*P* < 1e−16, *r*^2^ = 0.99) (Fig. 4), higher than the calculated increase of 1.0 mm/s using Equation (1). This discrepancy is likely due to the small boundary layer; velocity was measured in the working area of the flume away from the walls. Velocity was higher in the cryolite trials; for every 0.01 mL s^−1^ increase in flow rate, we observed a 2.58 mm/s (±0.35; 95% CI) increase in fluid velocity (*P* = 3.15e−10, *r*^2^ = 0.94) (Fig. 4). As a percentage of the mean, variability in velocity measurements was relatively consistent across flow rates in both the empty flume trials and cryolite trials (Table S2), with average % variability of 13.2 and 21.7%, respectively.

**Fig. 5:**
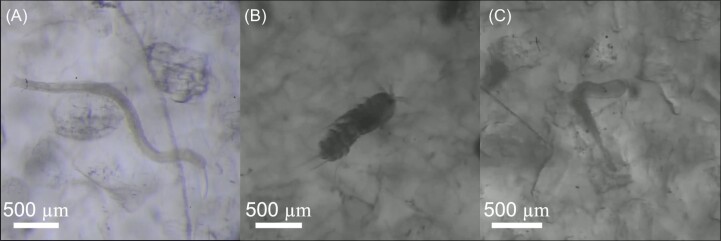
Faunal observations using the Meioflume. (A) A protodrilid (Annelida), moving through cryolite. (B) A gravid harpacticoid copepod in cryolite. (C) A flatworm in cryolite.

The difference in slopes between the cryolite trials and the theoretical data calculated using Equation (1) (Fig. 5) corresponds to a porosity within the cryolite trials that aligns well with literature values of marine sands. The slope ($\frac{{\bar{u}}}{Q}$) calculated from the cryolite trials is 2.58X the theoretical slope. Because flow rates and cross-sectional area of the tunnel are held constant between the theoretical and cryolite trials, Equation (2) dictates that this increase in slope must correspond to a decrease in the average porosity of the cryolite trials. An empty Meioflume, with its entire cross-sectional area available for fluid flow, can be thought to have a porosity of 100%, the 2.58 times reduction in porosity results in an average porosity of 38.75%. A review of published porosity values found that the porosities of natural marine sands ranged from 37.7 to 46.3% for packed to loose sands respectively (Curry et al. 2004). The average porosity of our cryolite trials falls toward the packed end of this range and indicates that caution must be taken to not overfill the Meioflume when preparing it for animal trials.

It is important to note that although we calibrated the mean velocity, flow velocity within granular media varies on small spatial scales. Flow through granular media follows the path of least resistance that varies with packing geometry (Stauffer and Aharony 1992). Packing the Meioflume with cryolite can lead to greater fluid velocities along the walls of the working area because the smooth surface of the walls leads to locally higher porosity due to non-uniform packing (Cohen and Metzner 1981). From a practical standpoint, this emphasizes the importance of adequate replication when using Meioflume to test hypotheses about the effects of flow velocity on behaviors, and observations made of animals directly adjacent to walls should be treated with caution. Finally, the calibration completed here (Fig. 4) is specific to the grain size of the cryolite used and the resulting porosity; other users should consider running their own calibration using each medium they intend to use in the Meioflume.

### Validating the Meioflume for observing meiofauna

To demonstrate the use of the Meioflume for studying the interstitial activities of meiofauna, we placed individual protodrilid annelids, platyhelminth flatworms, and harpacticoid copepods in Meioflumes packed with cryolite and recorded simple observations. Meiofauna were extracted using traditional decantation methods (Higgins and Thiel 1988; Giere 2009) from local sediments collected in Pelican Bay, Dauphin Island, AL, USA (30 14‘48.4‘ N, 88 07’19.8’ W), at ∼1-m water depth. The Meioflume was filled with seawater and packed with cryolite. Fauna were added by placing the target fauna in a small dish of cryolite and seawater, allowing them to migrate into the interstitial spaces, then gently pipetting the cryolite-seawater-fauna slurry into the working area through the loading notch, which was then sealed with putty. This method exploits the tendency of most meiofauna to adhere to sediment grains when disturbed, allowing them to be easily placed in the flume with the cryolite. Once primed, packed, and sealed, the Meioflume was placed on the stage of a dissection scope and connected to the syringe press. Animals were observed under no-flow conditions for 30 s, then exposed to 1 min of porewater flow at 0.005 mL s^−1^. Their responses were recorded using the camera system described in the calibration section above (Fig. 2). We observed five individual annelids and copepods and three individual flatworms. While a full behavioral analysis of the faunal responses observed is beyond the scope of this paper, we present three representative observations below.

A representative protodrilid annelid, platyhelminth flatworm, and harpacticoid copepod were all clearly visible within the Meioflume (Fig. 5A–C). The working area dimensions and cryolite allowed for detailed observations of behavior. Each animal could be clearly seen locomoting through the cryolite in the 30 s before flow began (Videos S1–S3) and then responding to flow in distinct ways. The protodrilid annelid, initially oriented with its head near the top of the Meioflume and its body extending down into the cryolite beneath it (Video S1: 1–30 s), turned and pointed its head toward the bottom of the Meioflume and began to move downward through the cryolite after the onset of flow (Video S1: 45 s). The harpacticoid copepod, which initially appeared to be moving between the cryolite and the lid of the Meioflume (Video S2: 0–30 s), began to burrow down into the cryolite almost immediately after flow began (Video S2 at 36 s). The platyhelminth flatworm moved through the interstitial spaces of the cryolite before flow began (Video S3; 1–30 s), briefly halted locomotion at the onset of flow (Video S3 at 30 s), then continued locomotion shortly after (Video S3 at 42 s), seemingly unperturbed.

There are several ways in which the Meioflume—cryolite system deviates from *in situ* environments that should be considered when designing an experiment. To enable clear visualization of fauna, the entire system is transparent that allows the working area to be flooded with light. Natural sediments, however, are dark, and many meiofauna are negatively phototactic (Giere 2009). Microscope lighting, from above or below, should be passed through a red light filter, and surrounding light intrusion should be as limited as possible. Additionally, our method for filling the Meioflume with cryolite does not account for grain packing. The extent to which a granular material is compacted determines the available space within the interstitial environment. As such, it is an important parameter in meiofaunal ecology and users should be careful to fill the Meioflume consistently across trials. Lastly, this system does not replicate the biotic factors of natural sediments. This system is intentionally abiotic so that fauna and biotic parameters can be introduced and studied in isolation, and this sterility should be considered when contextualizing behavioral observations.

## Implications and future developments

The Meioflume–cryolite system creates an interstitial environment where meiofauna are visible and porewater flow is manipulable. Here, we demonstrated steady-state flow, but the syringe pump control allows for programming flow velocity that can increase, decrease, or oscillate. Simulation of realistic porewater flow is limited by the lack of environmental data; development of methods to measure real-world flow through pore spaces would allow for direct relationships between meiofaunal behaviors and their natural environments. The Meioflume is a promising new tool in the field of meiobenthology and has the potential to greatly expand our understanding of meiofauna behavior in interstitial spaces, improving our ability to mechanistically connect the behaviors of meiofaunal individuals to their ecological effects. The low-cost design and construction of the Meioflume make it an accessible tool for researchers (see supplement for details).

The faunal observations made in the Meioflume currently rely on the use of cryolite, which can be difficult to source because of its limited availability; other options should be explored. Cryolite was initially mined in Ivigtut, Greenland, for commercial purposes (Josephson and Flessa 1972) but has not been produced since the late 1900s. It is now only sporadically available in small quantities from online gem sellers. A commercially available synthetic exists but does not work for this purpose due to high levels of impurities that render it opaque (Josephson and Flessa 1972). Without a transparent interstitial system, the Meioflume is of limited use in the study of interstitial meiofauna behavior, and widespread use of the Meioflume will likely require the adoption of a cryolite alternative. Currently, there are several potential substrates that could meet this need. Nafion is a synthetic fluoropolymer with a refractive index of 1.35 (Leis et al. 2005) that has been used as a transparent substrate in studies observing behavior and growth of microbes and root systems (Leis et al. 2005; Downie et al. 2012, 2014; O'Callaghan et al. 2018). Nafion is a superacid, requiring extensive chemical washing before it is safe for biological use (Downie et al. 2012), and its refractive index (1.35) is not an ideal match to that of seawater (1.33), leading to diminished transparency at the millimeter to centimeter scale (Sharma et al. 2020). Despite these caveats, Nafion may still be a useful alternative to cryolite within the Meioflume system because of the Meioflume's shallow depth (1 mm) and Nafion's wide commercial availability. Hydrogels offer another potential alternative to cryolite. Hydrogels can be made into spherical gel balls that are produced by combining alginate and gellan gum and dripping the mixture into a solution of MgCl_2_ (Ma et al. 2019). These gel spheres can then be hydrated with seawater and used as a transparent medium in the Meioflume. Hydrogels are inexpensive to produce, are almost completely transparent, and produce little optical distortion (Ma et al. 2019). Finally, as 3D printing technology continues to improve, resin printers could offer a novel method for producing transparent interstitial environments. New resin printers can produce 3D models with details as fine as 10 µm. At this resolution, models of stacked sand grains could be computer generated and printed using clear resin. Many 3D printing materials have limited biocompatibility, however, and exhibit high levels of toxicity to small organisms (Oskui et al. 2016). While these materials are not currently a viable option for direct use in biological experiments, such methods may become useful in the future if toxicity is reduced. Alternatively, the inverse approach could be taken, and models of interstitial space could be 3D printed, then used to cast models out of transparent materials with refractive indexes more similar to seawater, like the silicone polymer PDMS (RI = 1.43) (Wang et al. 2014).

This experimental system could easily be modified to address many questions in meiofaunal ecology such as organismal response to food availability, predator cues, chemical pollutants, or abiotic stressors such as oxygen availability, rising temperatures, or increased pH. As meiofauna are increasingly considered for using as indicators of environmental health and anthropogenic impacts (Zeppilli et al. 2015), understanding how these animals respond to abiotic shifts in the environment could provide valuable insights into their utility as indicator taxa. The Meioflume was designed to expose individuals to instantaneous cues, e.g., hydromechanical cues of changing water velocity or dissolved chemical cues, but it could also be used to assess sublethal responses to long-term exposure, e.g., of chemical pollutants on locomotory ability.

## Supplementary Material

obae016_Supplemental_Files
