# Supplementary material for: The Meioflume: A New System for Observing the Interstitial Behavior of Meiofauna
Source: Integr Org Biol. 2024 May 25;6(1):obae016. doi: 10.1093/iob/obae016 (PMC11177882; doi:10.1093/iob/obae016)
Supplement: obae016_Supplemental_Files [file obae016_supplemental_files.zip › Ballentine_Supplement.docx]

**Narrative design process**

In developing the Meioflume, we designed, tested, and modified 5 distinct versions, improving the functionality and usability with each version. We outline the different versions here, emphasizing what we learned through the process.

Version 1 (V1) of the Meioflume had a short, wide working area (Supplemental Fig 1 V1; Table 1) that tapered at both ends to a small opening in the bottom of the flume (Supplemental Fig 1 V1). These openings enabled fluid movement through the working area, but also allowed cryolite and fauna to be washed out of the flume. Packing and maintaining a static bed of cryolite in this model was not possible and animals frequently escaped through either end, severely limiting its utility.

Version 2 (V2) was designed to address the loss of cryolite and fauna by significantly extending the length of the working area (Supplemental Fig 1 V2). We reasoned that by extending the length, the small amount of cryolite lost at the excurrent end of the flume would be negligible to overall packing dynamics and that fauna would be less likely to encounter either fluid port. This proved ineffective and both cryolite and fauna were frequently washed out of the flume. We resolved this (Supplemental Fig 1 V2) by gluing 100-µm mesh barriers (Supplemental Fig 1 V2) at both ends of the working area. This modification effectively retained cryolite and animals, but the modified V2 was difficult to set up and use. The extended working area made locating animals within the flume time consuming, and achieving an effective seal was challenging. V2 sealed using a greased acrylic lid that bolted to the body of the Meioflume (Supplemental Fig 1 V2). Placing the lid, attaching the bolts, and balancing the torque was time consuming, and achieving a water-tight grease seal in the presence of cryolite grains was challenging. If any cryolite became caught between the body of the Meioflume and the lid, or if the bed was not perfectly level, the grease seal would fail, increasing preparation time and the likelihood of losing the animal within the flume. Additionally, the mesh barriers that retained cryolite and fauna in V2 were difficult to fit and secure, often extending higher than the walls of the working area, making it harder to seal the flume.

Version 3 (Supplemental V3) of the Meioflume had a working area with a shorter length and depth to reduce the time required to locate animals. Reducing the depth of the Meioflume also resulted in improved quality of animal observations as the reduction in cryolite and water between the specimen and microscope objective improved image clarity (Supplemental Fig 1 V3). The bolted acrylic lid was replaced with a standard glass microscope slide, sealed with a 1-mm-thick, clear silicone gasket, and held in place with four standard 2.54-cm binder clips (Supplemental Fig 1 V3 with lid). The binder clips compressed the silicone gasket between the glass slide and the Meioflume, and created a quick, robust seal that was quickly assembled and more forgiving of stray grains of cryolite or uneven beds. Finally, the entry and exit ports were moved from in-plane to below the working area, allowing the mesh fences in V2 to be replaced in V3 by 100 µm mesh covers that blocked the fluid entry and exit ports directly. To fully cover the entry ports in the bottom of the flume, the ports had to be moved away from the terminal ends so there was sufficient space behind the port to attach the mesh barrier. V3 successfully reduced the time to locate animals, improved observation quality, and was easier to seal than V2, but dye tests revealed that it did not generate porewater flow across the full width of the working area. When dye entered the working area through the entry hole, it formed a narrow stream of dye that crossed the working area and exited through the opposite hole without expanding to fill the width of the flume.

Version 4 (V4) addressed this problem with a two-piece design with a lower plate where fluid enters the device and expands to the width of the working area, and an upper plate which housed the working area and mesh covered openings of the same width (Supplemental Fig 1 V4). The acrylic plates were milled separately, then bonded using WELD-ON acrylic #4 acrylic bonding agent. Fluid enters V4 through one of two quick-turn tube couplings located on either end of the device (Supplemental Fig 1 V4) and into a triangular flow-widening chamber beneath the working area (Supplemental Fig 1 V4). Within the flow-widening chamber, the jet of incoming fluid gradually expands to the width of the overlying working area. Fluid then flows up through a 100 µm mesh screen (Supplemental Fig 1 V4), then into and across the working area. V4 reduced the length and width of the working area to be rapidly scanned at 20x magnification and generated the desired flow across nearly the entire width of the working area for the length of the device (Supplemental Fig 4 A-D). The glass slide, gasket, and clip sealing method used in V3 was retained in V4 (Supplemental Fig 1 V4 with lid), but we used this version enough to develop better ideas for sealing and packing the working area. Packing V4 required a cryolite seawater slurry be pipetted into the working area, then leveled with a spatula before the addition of fauna. If the bed was underfilled, the result was a loosely packed chamber with empty head space near the top of the working area. If the bed was overfilled, which occurred more often, the lid and gasket would compress the bed, reducing pore space and frequently trapping or crushing animals. V4 was fully usable in this configuration but required a substantial amount of training and practice to use effectively.

Version 5 (final version) (Fig 1 A & B) has the same dimensions as V4, containing a 28.57 x 10.16 x 1 mm working area with a cross sectional area of 0.1 cm^2^. The primary modification to the final version is the sealing method. We move back to a bolted, acrylic lid, but the lid is now attached and sealed to an empty, clean Meioflume, allowing for an easily achieved, robust seal. The lid seals over the entire working area, but at one end, a notch in the working area extends up past the fluid entry port and the edge of the lid (Fig 1 B). Cryolite and fauna are added to the working area through this notch, which is then sealed with a silicone plug. This method has the advantage of a robust seal that is free of contaminants, does not need to be disassembled between trials (cryolite and fauna can be pumped out through the notch), and, because the lid is in place before adding the cryolite, any packing that occurs is due to gravity rather than the compression from the lid. This small change to the final version produced substantial improvements to the ease of use and consistency of the Meioflume and greatly increased the speed at which experimental trails could be completed. Finally, in the final version, the quick-turn tube couplings were moved from the terminal ends of the device to the surface of the upper plate (Fig 1 A& B). This change in location improved the accuracy of the fabrication process, by removing the need to perfectly align the device when drilling holes on either end, and did not affect the fluid dynamics. A full CAD file and parts list for the final version of the Meioflume can be found at (<https://github.com/Will-Ballentine/Meioflume_Designs.git> ) (Supplemental table 3).To use the final version of the Meioflume, the quick-turn tube couplings are both equipped with three-way stop cock valves, attached to the syringe press and a waste seawater reservoir via tubing, and placed on the stage of a microscope for observation and recording (Fig 2).


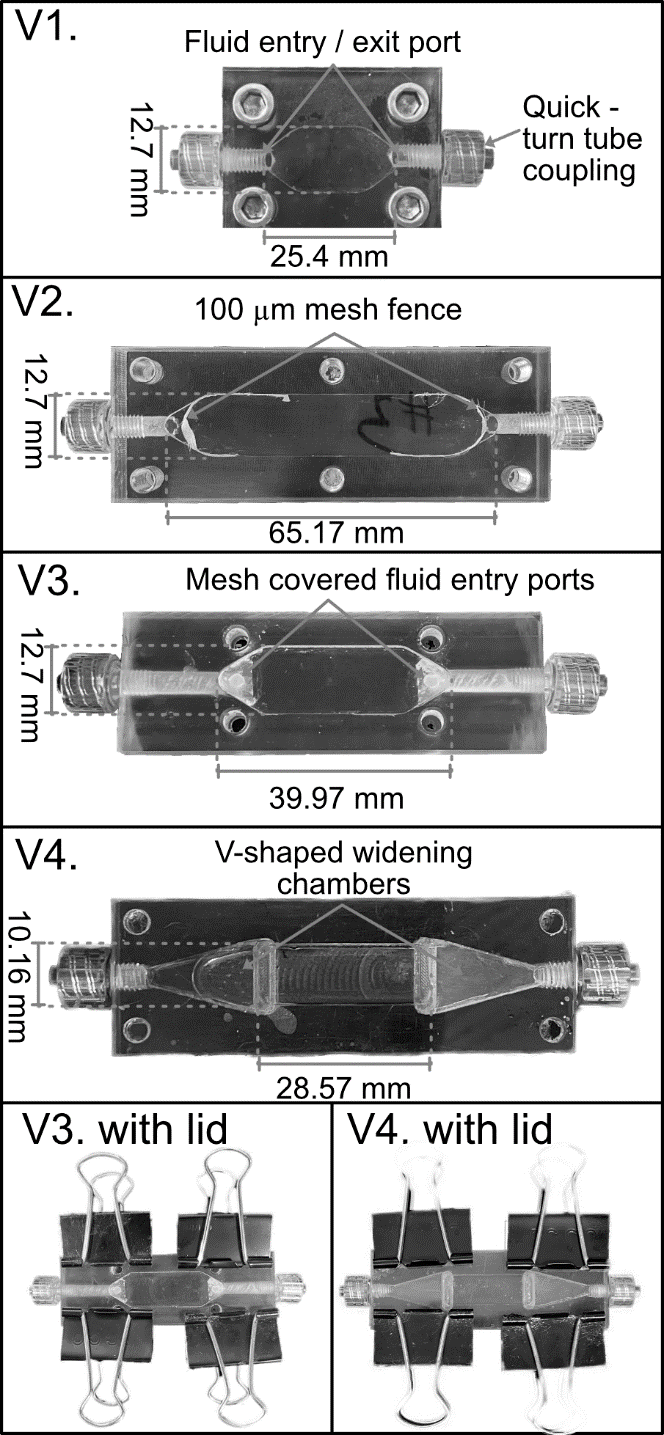


Supplemental Figure 1: Previous iterations of the Meioflume illustrating key design changes. **V1.** The original Meioflume with a convex, short, deep working area, no fauna or grain retention strategy, and a bolted acrylic lid. **V2.** The second Meioflume with a much longer, convex working area and a 100 µm mesh fence at either end to retain cryolite and fauna. **V3.** The third Meioflume design with a convex working area that was shorter, narrower and shallower than previous designs with 100 µm mesh covering the flow entry and exit ports (Lid and clips shown in **V3. with lid**). This was the first model capped using a glass microscope slide and a silicone gasket. **V4.** The fourth Meioflume design featured a rectangular working area that was shorter than the third design. Fluid entered this working area by first entering a V-shaped flow – widener beneath the working area and percolated up through a 100 µm mesh cover into and across the working area (shown with lid in **V4. with lid**).

Supplemental Video 1: Protodrilid in the Meioflume. The protodrilid annelid, initially oriented with its head near the top of the Meioflume and its body extending down into the cryolite beneath it (1-30 s), turned and pointed its head toward the bottom of the Meioflume and began to move downward through the cryolite after the onset of flow (1: 45 s)

Supplemental Video 2: Harpacticoid copepod in the Meioflume. The harpacticoid copepod, which initially appeared to be moving between the cryolite and the lid of the Meioflume (0-30 s), began to burrow down into the cryolite almost immediately after flow began (2: 36 s).

Supplemental Video 3: Platyhelminth flatworm in the Meioflume. The platyhelminth flatworm moved through the interstitial spaces of the cryolite before flow began (1-30 s), briefly halted locomotion at the onset of flow (30 s), then continued locomotion shortly after (42 s), seemingly unperturbed.

| It. | W.A. geom | W.A. length mm | W.A. width mm | W.A. depth mm | Lid material | Sealing mechanism | Grain/fauna retention | Flow Development |
| --- | --- | --- | --- | --- | --- | --- | --- | --- |
| 1 | Hex | 25.4 | 12.7 | 1.6 | Acrylic | Grease/bolt | None | Convex W.A |
| 2 | Hex | 65.17 | 12.7 | 1.6 | Acrylic | Grease/bolt | Mesh fence | Convex W.A |
| 3 | Hex | 39.77 | 12.7 | 1 | Glass | Clips/gasket | Mesh entry | Convex W.A |
| 4 | Rect. | 28.57 | 10.16 | 1 | Glass | Clips/gasket | Mesh entry | V – shaped flow widener |
| 5 | Rect. | 28.57 | 10.16 | 1 | Acrylic | Grease/bolt + putty | Mesh entry | V-Shaped widener |

Supplemental table 1: Relevant design dimensions of Meioflume designs. It. Iteration W.A. Working area.

| Flow Rate (mL/s) | Fill | Theoretical velocity (mm/s) | Mean Velocity (mm/s) | Standard  Deviation | Percent Variability |
| --- | --- | --- | --- | --- | --- |
| 0.0005 | Empty | 0.05 | 0.0471 | 0.00851 | 18.06 |
| 0.001 | Empty | 0.1 | 0.0946 | 0.0137 | 14.46 |
| 0.01 | Empty | 1.0 | 1.16 | 0.084 | 7.21 |
| 0.0005 | Cryolite | 0.05 | 0.110 | 0.0169 | 15.43 |
| 0.001 | Cryolite | 0.1 | 0.219 | 0.0506 | 23.08 |
| 0.01 | Cryolite | 1.0 | 2.55 | 0.686 | 26.88 |

Supplemental Table 2: Average fluid velocities for each flow rate and fill. Theoretical velocity calculated by dividing the flow rate by the cross section area of the working area (0.1 cm^2). Percent variability is the standard deviation calculated as a percent of the mean.

| Item | Count | Cost | Source | Part Number |
| --- | --- | --- | --- | --- |
| 0.25 ” Clear acrylic stock (body) | 1 | $18.38 | McMaster-Carr | 8560K354 |
| 1/16 “ clear acrylic stock (lid) | 1 | $5.61 | McMaster-Carr | 8560K171 |
| Plastic Quick-Turn Tube Coupling, Plugs, 10-32 Unf Male | 10 pack | $9.38 | McMaster-Carr | 51525K431 |
| Plastic Quick-Turn 3-Way Stopcock Valve | 2 | $2.66 | McMaster-Carr | 7033T24 |
| 316 Stainless Steel Socket Head Screw, 6-32 Thread Size, 1/4" Long (4x) | 25 pack | $3.37 | McMaster-Carr | 92185A144 |
| Dow Corning High Vacuum Grease | 5.3 oz tube | $40.00 | Amazon | NA |

Supplemental Table 3: Required stock materials and pricing. Price estimates reflect pricing at time of publication. Does not include syringe press.
